# Protective effects and potential mechanisms of fermented egg-milk peptides on the damaged intestinal barrier

**DOI:** 10.3389/fnut.2022.1068877

**Published:** 2022-12-07

**Authors:** Siwen Lyu, Qi Yang, Xuehui Duan, Xuanting Liu, Zhiyang Du, Xiaomin Shang, Menglei Xu, Jingbo Liu, Fengguang Pan, Ting Zhang

**Affiliations:** ^1^Jilin Provincial Key Laboratory of Nutrition and Functional Food, College of Food Science and Engineering, Jilin University, Changchun, China; ^2^State Key Laboratory of Supramolecular Structure and Materials, Jilin University, Changchun, China

**Keywords:** peptides, intestinal barrier, zonula occludens-1, mucin, bioinformatics

## Abstract

**Introduction:**

Fermented egg-milk peptides (FEMPs) could enhance the colon-intestinal barrier and upgrade the expression of zonula occludens-1 and mucin 2. Besides, the underlying biological mechanism and the targets FEMPs could regulate were analyzed in our study.

**Methods:**

Herein, the immunofluorescence technique and western blot were utilized to evaluate the repair of the intestinal barrier. Network pharmacology analysis and bioinformatics methods were performed to investigate the targets and pathways affected by FEMPs.

**Results and discussion:**

Animal experiments showed that FEMPs could restore intestinal damage and enhance the expression of two key proteins. The pharmacological results revealed that FEMPs could regulate targets related to kinase activity, such as AKT, CASP, RAF, and GSK. The above targets could interact with each other. GO analysis indicated that the targets regulated by FEMPs could participate in the kinase activity of the metabolic process. KEGG enrichment revealed that the core targets were enriched in pathways related to cell apoptosis and other important procedures. Molecular docking demonstrated that FEMPs could bind to the key target AKT *via* hydrogen bond interactions. Our study combined the experiment *in vivo* with the method *in silico* and investigated the interaction between peptides and targets in a pattern of multi-targets and multi-pathways, which offered a new perspective on the functional validation and potential application of bioactive peptides.

## Introduction

To protect the body from the environment, the intestinal tract—one of the largest luminal interaction areas—contributes more to the body. The intestinal barrier was reported to be vital in regulating the immune system, improving nutrition absorption, and maintaining intestinal health (1). Among the treatments for intestinal diseases, many potential therapies aimed to develop new drugs to protect the intestinal barrier and repair damage to the intestinal mucosa. It is essential to keep the intestinal barrier intact and healthy. The weakness of the intestinal barrier has been linked to fat, bile acids, emulsifiers, and gliadin (2). Damage to the intestinal barrier could cause abdominal pain, diarrhea, and indigestion. As the previous paper reviewed, the intestinal barrier might drive a wide association with other chronic metabolic diseases (3), such as upper gastrointestinal diseases, inflammatory bowel disease (IBD), celiac disease, and non-alcoholic fatty liver disease. In the body of patients with IBD, the damaged intestinal barrier could upgrade the expression of pro-inflammatory cytokines, such as tumor necrosis factor-α (TNF-α) and interleukin−1β (IL-1β), which exacerbated the inflammatory response (4, 5). Thus, the intestinal barrier is a crucial point linked to IBD. Research on celiac disease indicated that increased intestinal tract permeability might cause negative secondary effects, such as vicious cycles of intestinal cell damage (6). In addition to the diseases listed above, the intestinal barrier is also related to obesity and diabetes.

A reference has confirmed that the changing of the intestinal barrier might be a pathological cause of obesity. A type of intestinal microbial metabolite called short-chain fatty acids was found to regulate intestinal dysfunction and ameliorate obesity (7). Another study reported that the disruption of the intestinal biological barrier could cause the aggregative symptoms of diabetes (8). Based on the association between the intestinal barrier and other metabolic diseases, it is of great significance to maintain stability and repair the intestinal barrier. Different kinds of tight junctions, mucosa, and numerous mucosal epithelia, which constitute the intestinal barrier (3), were beneficial in enhancing the powerful protective function of the intestinal. Zonula occludens−1 (ZO-1), one of the vital tight junctions (TJs), was reported to have an integral effect on intestinal barrier function and mucosal permeability (9). Mucin 2 (MUC-2), a vital element of mucus components, is secreted by the intestinal goblet cells (10). Therefore, these two proteins have become one of the indicators to determine whether the intestinal composition is complete and whether the barrier function is sound.

On the basis of the consequences of the intestinal barrier on the health of the body, people have become increasingly interested in functional and effective food ingredients. Protein derived from egg white was claimed as the feature of repairing the damaged intestinal barrier (11). Bioactive peptides, obtained under the effects of hydrolysis and fermentation (12), have the potential to be functional ingredients to enhance the intestinal barrier and cure intestinal diseases. Peptides derived from eggs have been widely reported as having biological functions, and they could be functional ingredients to enhance body health (13). As we expected, egg white peptides were reported for their effect on the intestinal wall, which represented intestinal barrier repair and gut microbiota regulation. Ge has demonstrated the alleviative effect of egg white peptides on the colon in colitis mice. The results confirmed that egg white peptides could repair the mucosa and intestinal structure of the damaged tissue (14). Our previous research also investigated the protective function of fermented egg milk on colitis induced by dextran sulfate sodium (DSS) in mice (15). However, the underlying mechanism and potential targets of the fermented egg-milk peptides' (FEMPs) action on the damaged intestinal barrier need further research.

In extensive research on the mechanism of bioactive peptides regulating the intestinal barrier, researchers mostly mentioned a single target and pathway. Unlike the traditional idea, network pharmacology, a novel, and powerful tool provide evidence for the possible mechanism of action of multi-target and multi-pathway disease regulation (16). It has been utilized in the research of colonic diseases (17, 18). Molecular docking is a justified and proven instrument to analyze the interaction between receptors and ligands. Recently, molecular docking has been widely utilized in the field of computer-aided binding for predicting peptides with bioactive functions (19). Herein, we used the idea and methods of network pharmacology to analyze the possible and potential targets FEMPs could affect, and the interaction and relationship between targets were further investigated. Gene ontology (GO) analysis and Kyoto Encyclopedia of Genes and Genomes (KEGG) enrichment analysis, as the emphasis of network pharmacology, were often used to carry out and illustrate the function of the core targets.

In our study, the DSS-induced colitis mouse model was applied to evaluate the repair of the intestinal barrier. The ZO-1 and MUC-2 were focused on as the factors. Besides, the potential pharmacological mechanism under the FEMPs effect on the intestinal barrier was performed *via* the idea of network pharmacology and the method of molecular docking. This study combines *in vivo* assessment with *in silico* evaluation, which could provide a new perspective for intestinal barrier enhancement and colonic disease treatment and broaden the horizons of egg products' functional applications.

## Materials and methods

### Materials

FEMPs were acquired in our lab according to the previous method. DSS (Mw, 50000 Da) was purchased from Sigma-Aldrich Trading Co., Ltd. (Shanghai). Biodewax and clear solution, 4′,6-diamidino-2-phenylindole (DAPI), and ethylenediamine tetraacetic acid (EDTA) (pH 8.0) antigen retrieval solution were bought from Wuhan Servicebio Technology Co., Ltd. Ethanol was purchased from Sinopharm Chemical Reagent Co., Ltd, and BCA protein quantitative detection kit, β-actin, and TBS buffer solution were bought from Wuhan Servicebio Technology Co., Ltd.

### Animal experiments design

A total of 60 Balb/c mice (male, 8 weeks old, SPF level) were obtained from Beijing Charles River Co., Ltd (Beijing, China) and housed in the lab in the Animal Model Laboratory Building at Jilin University. The housing condition was adjusted to 20–23°C of temperature, 40–70% of humidity, and a light-dark cycle of 12 h per day. Before the beginning of the experiment, the mice were made to acclimate to the environment and were given food and water freely. All the animal procedures in the whole experiment were implemented in accordance with the guidelines for laboratory animal care and use at Jilin University. The animal experiments were reviewed and confirmed by the Jilin University animal ethics committee (Approval No. 20200483). The entire experiment's anesthetic-required procedures were performed under the influence of isoflurane.

For the allocation of the 60 mice (22–24 g), the five groups were settled, which were named CK (the short name of control check), CK + FDP (control check and drink FEMPs freely), DSS + FDW (dextran sulfate sodium and drink water freely), DSS + GP (DSS and gavage FEMPs), and DSS + FDP (DSS and drink FEMPs freely). The integral experiment period was divided into the intestinal damage-making period (period 1, days 0–7) and the treatment period (period 2, days 7–21). During period 1, the DSS, the DSS + GP, and the DSS + FDP groups were made to drink 3% DSS solution instead of making them drink water to run the intestinal-damaged colitis model. Meanwhile, the CK and the CK + FDP groups were provided with free food and water. During period 2, the DSS + GP group was asked to obtain 200 mg/kg/day FEMPs by gavage. The CK + FDP and the DSS + FDP were required to drink FEMPs freely for 4 h and drink water for the other 20 h per day. The DSS + FDW group was asked to drink water. All the groups were given free mouse food, and the body status of all the mice was recorded during the experiment periods. The mice were euthanized according to animal procedures and guidelines. The colons were harvested and weighted, then cryopreserved at −80 °C.

### Immunohistochemistry staining

Incubate sections were deparaffinized and rehydrated with xylene and absolute ethanol before being placed in an EDTA antigen retrieval buffer with a pH value of 8.0. Maintained at a sub-boiling temperature for 8 min, we washed the sections three times with PBS (pH 7.4). Then, 3% BSA was added here to cover the marked tissue and block non-specific binding in 30 min. After that, the primary antibody and the secondary antibody were provided. DAPI counterstain was carried out in the nucleus, threw away the liquid carefully, then the slips were covered with the anti-fade mounting medium. The incubated sections were detected and envisioned by fluorescent microscopy.

### Western blot analysis

The western blot analysis was developed under the protocol of a previous study with some modifications (20). The key proteins (ZO-1 and MUC-2) in colon tissue lysates were removed by an SDS-polyacrylamide gel and transferred electrophoretically to a polyvinylidene difluoride (PVDF) membrane. We placed the transferred membrane into the TBST incubation tank for a quick wash before blocking the membrane for 30 min with a blocking buffer (5% milk) at room temperature. After that, the membrane was incubated with appropriate dilutions of primary antibodies. The membrane was incubated with a dilution of 1:5000 of conjugated secondary antibody in blocking buffer at a temperature condition in a room for 30 min. After that, the film was washed three times (5 min each time) with TBST. The acquired pictures were obtained with darkroom development techniques for chemiluminescence. The performance of ECL was in accordance with the manufacturer's description, adding ECL reagents for 1–2 min at room temperature, and the WB images were captured under the various times of exposure.

### FEMPs information preparation and target gene prediction

The sequence information for FEMPs was based on the database in our lab and silicon digestion. The ExPASy PeptideCutter program, a database available at https://web.expasy.org/peptide_cutter/, was used here to collect the FEMPs' information. All the proteins and peptides in fermented egg milk and the sequences of LC-MS/MS results were collected based on our previous study (15) and uploaded into the PeptideCutter database. Pepsin and trypsin were selected as the digestive enzymes to perform the digestion process. All sequences of FEMPs were uploaded to the Pharmmapper platform at http://www.lilab-ecust.cn/pharmmapper/index.php. To screen the targets, FEMPs could be regulated (21). The targets were recorded according to the Fit score. The Uniprot platform (http://www.uniprot.org/) was utilized here to filter the Homo targets. Intestinal barrier-related targets were obtained from the GeneCard database (https://www.genecards.org). Besides, the targets obtained from references linked to intestinal health were used to supplement the targets set.

### Construction of the protein-protein interaction (PPI) network

To clarify whether FEMPs could regulate the relationship between targets, we established and analyzed a PPI network of the targets. The STRING database (http://string-db.org/) (22), a powerful platform for analyzing current and possible protein-protein interactions, was used to establish functional protein association networks based on computational prediction, knowledge conversion between different organisms, and interactions aggregated with other related databases. The top 53 targets were input to the STRING database to analyze the potential co-interaction, and the STRING database depicted the figure of the PPI network.

### GO and KEGG enrichment analysis

To understand the underlying effect and mechanism of intestinal barrier functions of the core targets, we performed the GO and KEGG enrichment analysis based on the Metascape software (https://metascape.org) (23). In the custom analysis process, homo sapiens was set as the analysis species. GO biological processes (BP), GO cellular components (CC), and GO molecular functions (MF) were ticked in this part. The parameters of the analysis were shown: Min overlap, 3. *P*-value cutoff, 0.01, Min enrichment, 1.5. All the results were visualized by a free online bioinformatics database (http://www.bioinformatics.com.cn/).

### Pathway regulation

FEMPs' regulation of the pathway was analyzed by Metascape software, and the related genes were marked by the website of the KEGG pathway (https://www.genome.jp/kegg/pathway.html). The targets FEMPs could influence in the pathway were marked in a different color, and the figure was visualized *via* Adobe Illustrator software.

### Molecular docking

Based on the computer calculation, molecular docking, a considerable tool widely used to reveal the relationship and interaction between receptors and ligands, was used here to analyze the action of FEMPs on the targets. The calculation has been extensively utilized and confirmed to predict the interaction energy between different molecules. In the current research, molecular docking was performed based on the Autodock Tools and Autodock Vina software. Based on the node degree analyzed by the PPI network (Supplementary Table S1), the AKT showed the highest degree. Herein, the AKT target was selected as the receptor to perform the molecular docking process, and the FEMPs were developed as ligands. All the files of receptors and ligands with the PDBQT format were set as the input files for molecular docking experiments. The crystal structure of AKT (1UNQ, PDB doi: 10.2210/pdb1UNQ/pdb) was downloaded from the database of the RCSB Protein Data Bank (http://www.rcsb.org/pdb), and the organism of the protein was selected as “Homo.” All the molecules of water were excluded from the crystal structure of AKT (1UNQ) to prepare for the molecular docking process. The binding site detection was according to the reference. In this study, a grid box of 25 × 25 × 25 Å was established at the surrounding binding site location, and the grid was generated suitably for peptide docking. The flexible style of the peptides was selected. The hydrogen bonds and cooperative interactions between the AKT residues (1UNQ) and ligands were visualized and analyzed.

## Results

### FEMPs enhance the expression of ZO-1 and MUC-2

Previous references have confirmed the significance and effect of TJs in the occurrence and preservation of UC (24, 25). ZO-1, a kind of vital peripheral membrane protein in the colon, maintained the junction network's relationship. It could enhance the colon barrier by linking claudins, occludin, and other proteins. MUC-2 was the vital component of mucus, with the effect of forming an indispensable barrier against the pathogen in the tissue of the intestine. Besides, MUC-2 was reported to preserve the mucus layers of colon tissue. The content of MUC-2 was also related to the number of intestinal goblet cells (26), which might benefit the immune function of the colon. Our study investigated the expression of these two vital proteins by immunohistochemistry staining and western blotting.

**Figure 1:**
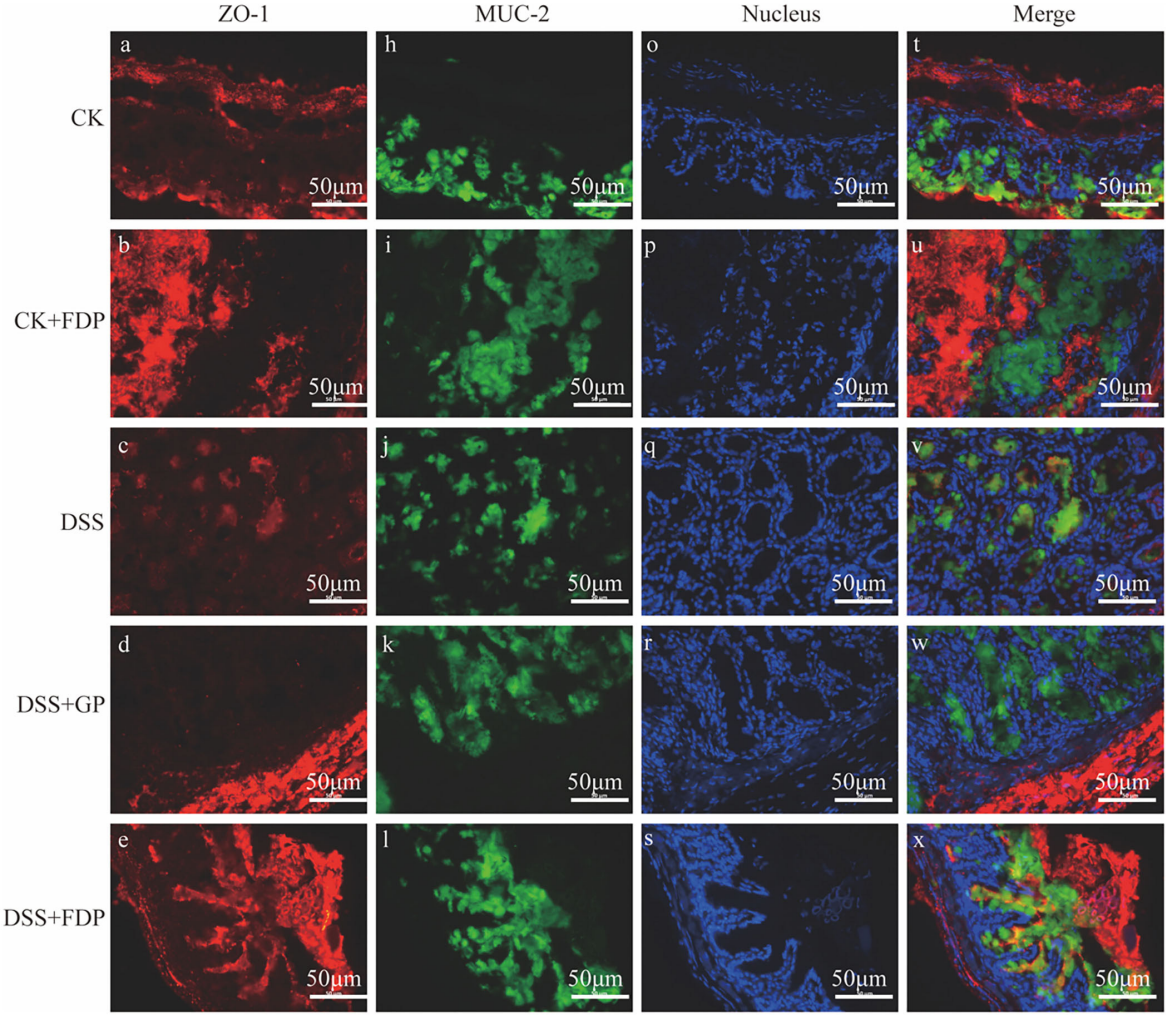
Effects of fermented egg-milk peptides (FEMPs) treatment on the expression of ZO-1 (red) and MUC-2 (green) in colitis mice. The pictures were observed by a fluorescence microscope of 400×. CK, control check. CK + FDP, control check and drink FEMPs freely. DSS + FDW, DSS and drink water freely. DSS + GP, DSS and gavage FEMPs. DSS + FDP, DSS and drink FEMPs freely. The different labels a-x represented for the different samples.

The results of immunohistochemistry staining revealed the two vital proteins locally. As Figure 1 depicts, the nucleus in intestine epithelial cells was blue, ZO-1 was red, and MUC-2 was green. The intensity and area of immunofluorescence staining represented the expression levels of the two proteins. Figure 1 shows that in the CK and CK+FDP groups, ZO-1 and MUC-2 were distributed around the nucleus, which meant the normal expression of the two proteins. The intestinal structure was complete and sound, and the intestinal barrier function could be normal and powerful. By contrast, DSS treatment induced an obvious loss in the content of ZO-1 and a considerable decrease in the expression of MUC-2, which showed that the gut structure was damaged and weak. Without the protection of the intestinal barrier, the function of the gut might be influenced. FEMP treatment could repair the colonic damage induced by DSS and enhance the expression of two vital proteins. In addition, the expression of ZO-1 and MUC-2 was upregulated. As the picture depicts, the fluorescence intensity of ZO-1 and MUC-2 was enhanced, which indicated that the location and expression of these two significant proteins were restored.

**Figure 2:**
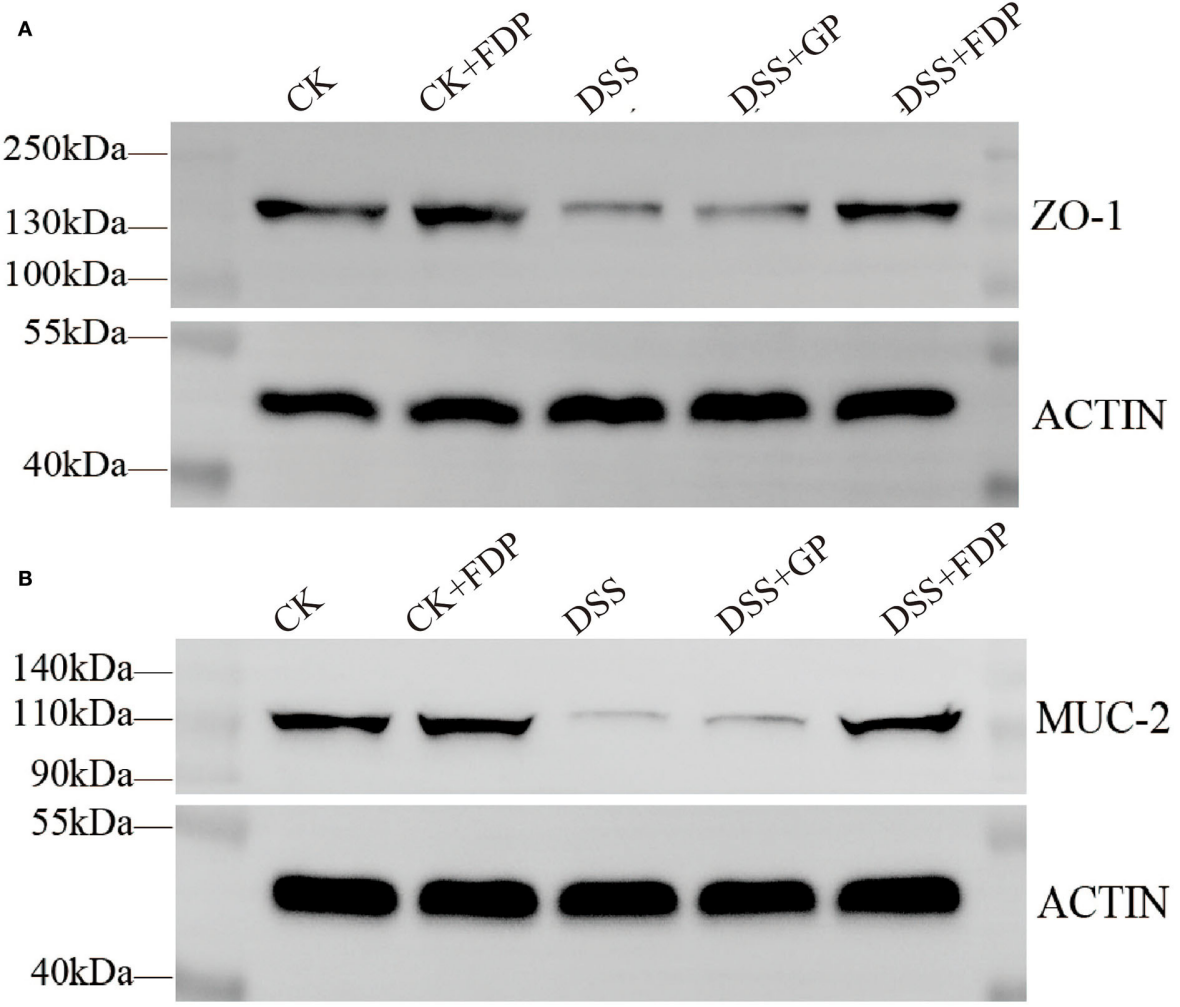
Western blot analysis of tight junction proteins. Zonula occludens−1 (ZO-1) **(A)** and Mucin 2 (MUC-2) **(B)** from mice colon tissues. CK, control check. CK + FDP, control check and drink FEMPs freely. DSS + FDW, DSS and drink water freely. DSS + GP, DSS and gavage FEMPs. DSS + FDP, DSS and drink FEMPs freely.

To further determine the relationship between the observed proteins and FEMPs treatment in experimental colitis, an experiment with the western blot was performed in our study. The proteins were extracted from the colons of the mice. As shown in Figure 2, both ZO-1 and MUC-2 displayed a decreasing trend in the DSS group, which meant these two proteins weakened in the colitis body. The proteins in the DSS+GP group and the DSS+FDP group were restored, which indicated that FEMPs might benefit from the increased levels of ZO-1 and MUC-2. Both the immunohistochemistry staining and western blotting revealed the positive effect of FEMPs on the damaged intestinal barrier.

### Analysis of the PPI network

**Figure 3:**
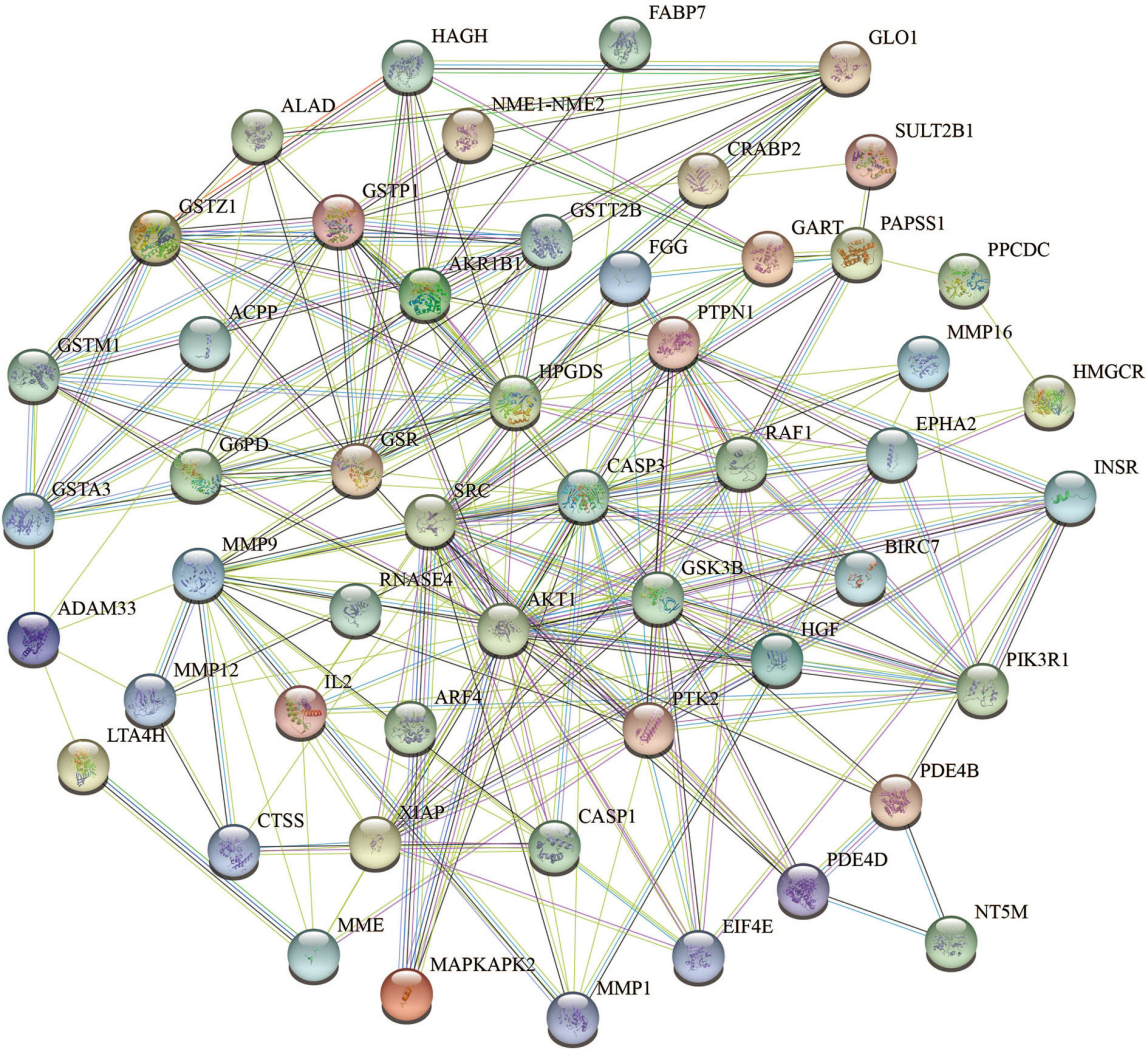
Protein-protein interaction (PPI) network of the targets could be regulated by fermented egg-milk peptides (FEMPs). The nodes represented for the targets, and the edges represented for the interactions between the targets.

To acquire information on targets that FEMPs could regulate, we developed target matching based on the Pharmmapper website. According to the network pharmacology method, a total of 52 targets (the top 52) were contained in the FEMPs targets database. The STRING website was applied to perform the PPI network. After that, a network containing 52 nodes and 223 edges was received, and the enrichment *p*-value of this network was 1.0e−16 (Figure 3). In the structure of the PPI network, the nodes represented the targets, and the edges represented the interactions. The more edges a node emerged, the more important role it would play in the network. Notably, the targets AKT1, CASP3, SRC, HPGDS, and MMP9 were connected with other targets, which revealed that these targets could generate more interaction and play a linkage role. Meanwhile, the node degree illustrated the same results that the above-mentioned targets have the potential to affect other targets (Supplementary Table S1).

To further demonstrate the information on interactions and functions of the PPI, the interaction analysis of the edges was calculated by the STRING database. The edges with a combined score larger than 0.90 are listed in Table 1. The results indicated that the interaction between CASP3 and XIAP, GLO1 and HAGH, INSR and PTPN1, PIK3R1 and SRC showed a strong relationship, with a combined score of 0.999. Besides, the co-interactions between PTPN1 and SRC, AKT1 and GSK3B, AKT1 and PIK3R1, and INSR and PIK3R1 revealed an intensive influence in the PPI network. All of these results demonstrated the potential interaction among the targets.

**Table 1:** The line information in protein-protein interaction network (PPI).

**Node1**	**Node2**	**Coexpression**	**Interaction**	**Combined score**	**Node1**	**Node2**	**Coexpression**	**Interaction**	**Combined score**
CASP3	XIAP	0.062	0.984	0.999	HGF	MMP1	0.067	0	0.952
GLO1	HAGH	0.145	0	0.999	CASP3	EIF4E	0	0	0.948
INSR	PTPN1	0.062	0.985	0.999	EPHA2	PIK3R1	0	0.316	0.943
PIK3R1	SRC	0	0.889	0.999	AKT1	INSR	0.052	0.182	0.942
PTPN1	SRC	0.086	0.875	0.997	PIK3R1	PTPN1	0	0.095	0.940
AKT1	GSK3B	0.049	0.934	0.996	RAF1	SRC	0	0.620	0.940
AKT1	PIK3R1	0.062	0.7	0.996	AKT1	CASP3	0	0.475	0.935
INSR	PIK3R1	0.062	0.895	0.995	GSR	HPGDS	0.064	0	0.933
PIK3R1	PTK2	0	0.875	0.995	RAF1	XIAP	0.053	0.859	0.931
PTK2	SRC	0.062	0.931	0.994	GSTP1	GSTZ1	0.064	0.161	0.929
AKT1	RAF1	0	0.880	0.992	CASP1	CASP3	0	0	0.928
BIRC7	CASP3	0.058	0.726	0.987	EPHA2	SRC	0.096	0	0.927
AKT1	SRC	0.098	0.683	0.985	HGF	PTPN1	0	0.056	0.924
AKT1	PTPN1	0.091	0.284	0.975	IL2	PIK3R1	0	0	0.920
HGF	MMP9	0.062	0	0.970	HGF	PIK3R1	0	0	0.919
MMP9	SRC	0.089	0	0.970	GSTT2B	HPGDS	0.065	0.147	0.914
AKT1	IL2	0	0	0.966	GSTZ1	HPGDS	0.064	0.161	0.913
HGF	SRC	0	0.056	0.963	GSTP1	GSTT2B	0.065	0.352	0.912
MMP1	MMP9	0.518	0	0.961	GSTM1	GSTZ1	0.064	0.161	0.908
AKT1	XIAP	0	0.759	0.955	FGG	PTPN1	0	0.103	0.907
EPHA2	PTK2	0.098	0.298	0.954	CASP1	EIF4E	0	0	0.906
CASP3	PTK2	0	0	0.953	PAPSS1	RAF1	0.049	0.061	0.903

### GO and KEGG enrichment analysis

**Figure 4:**
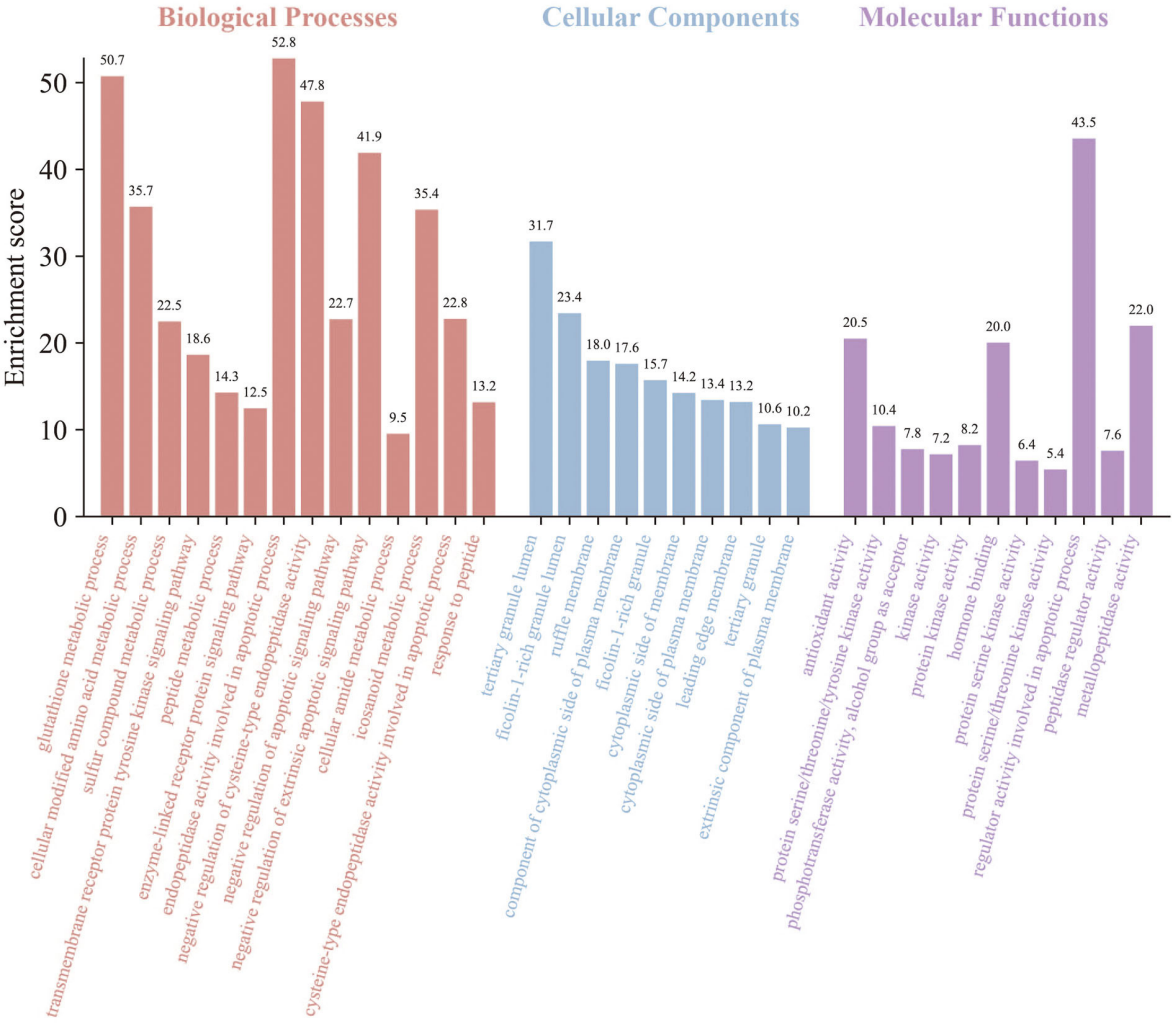
Gene ontology (GO) enrichment of key targets. Biological processes, cellular components, and molecular functions were mentioned. The enrichment scores were marked in the picture.

The GO and KEGG enrichment analyses of the above targets were carried out by Metascape software to further find out their potential biological functions. The parts of BP, CC, and MF were performed. The top 35 results (14 highly enriched in BP, 10 highly enriched in CC, and 11 highly enriched in MF) are shown in Figure 4. For BPs, the results indicated the core targets participated in the endopeptidase activity involved in the apoptotic process with an enrichment score of 52.8. Meanwhile, the glutathione metabolic process was enriched with an enrichment score of 50.7. The regulation of cysteine-type endopeptidase activity (with an enrichment score of 47.8) was shown here. Besides, the results also illustrated the process of response to peptides and the peptide metabolic process, which corresponds to the FEMPs. For the parts of CC, tertiary granule lumen, ficolin-1-rich granule lumen, and ruffle membrane were contained with enrichment scores of 31.7, 23.4, and 18.0, respectively. These results indicated that the targets could participate in the regulation of cell structures. We could observe from the MF enrichment results that the core targets could affect the regular activity involved in the apoptotic process with an enrichment score of 43.5, metallopeptidase activity with an enrichment score of 22.0, and protein serine/threonine/tyrosine kinase activity with an enrichment score of 10.4.

**Figure 5:**
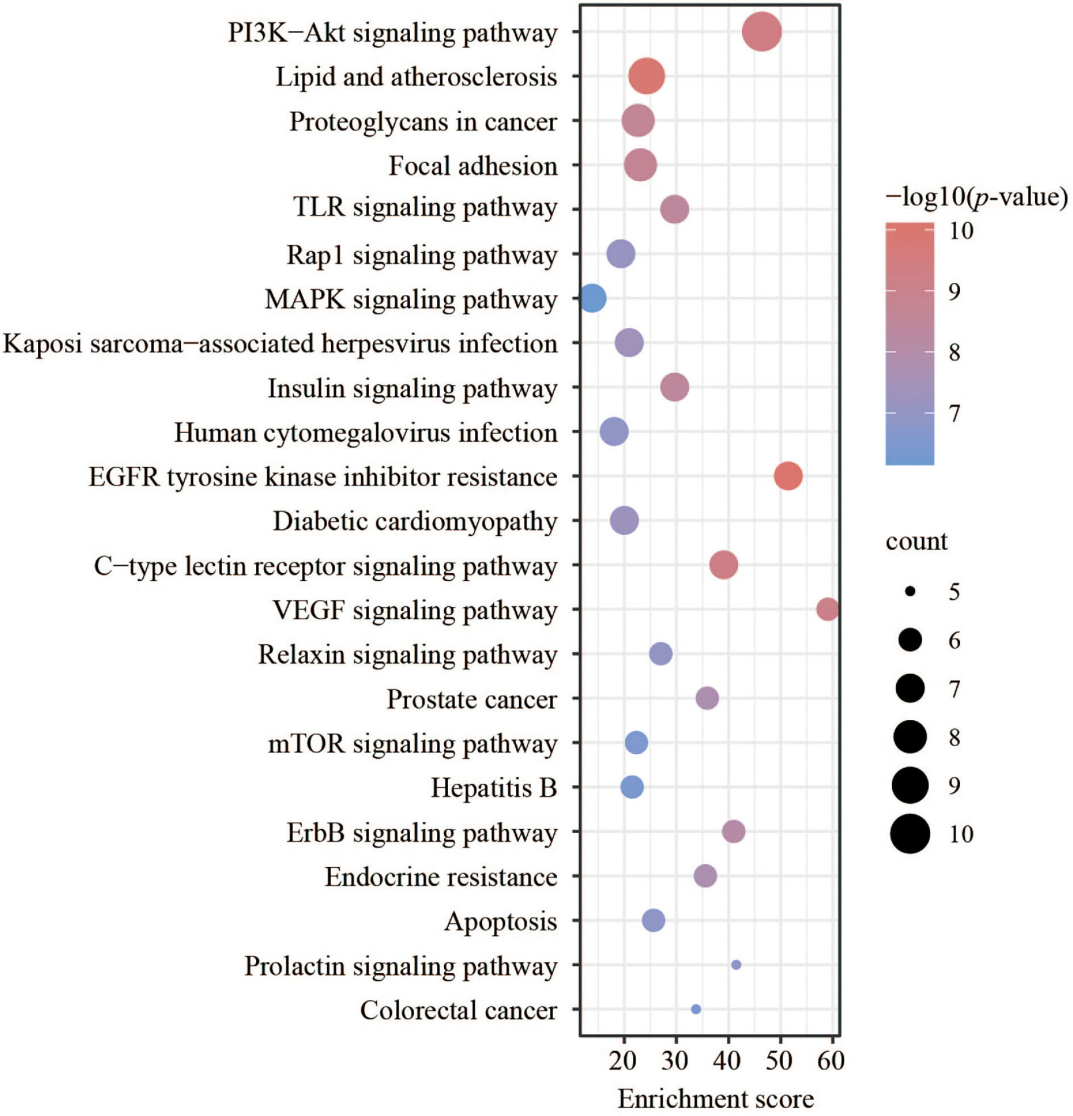
Kyoto Encyclopedia of Genes and Genomes (KEGG) pathway analysis of key targets.

For the KEGG pathway enrichment analysis, various signaling pathways related to cell proliferation, differentiation, morphogenesis, and apoptosis were analyzed. The results demonstrated that there were 23 pathways with a number < 5, as listed in Figure 5. The typical signaling pathway might influence the intestinal epithelial cells and intestinal barrier functions mentioned in this study. The PI3K-Akt signaling pathway, with a *p*-value of 4.41e−10, was enriched with the largest count of 10. These results showed that the targets could participate more in the PI3K-Akt signaling pathway. VEGF signaling pathway and EGFR tyrosine kinase inhibitor resistance were listed here with an enrichment score of 59.06 and 51.46, respectively. Meanwhile, many pathways related to intestinal health, such as the TLR signaling pathway (27), MAPK signaling pathway (28), and Rap1 signaling pathway, were enriched with a high enrichment score.

### Regulation of PI3K-Akt signaling pathway

**Figure 6:**
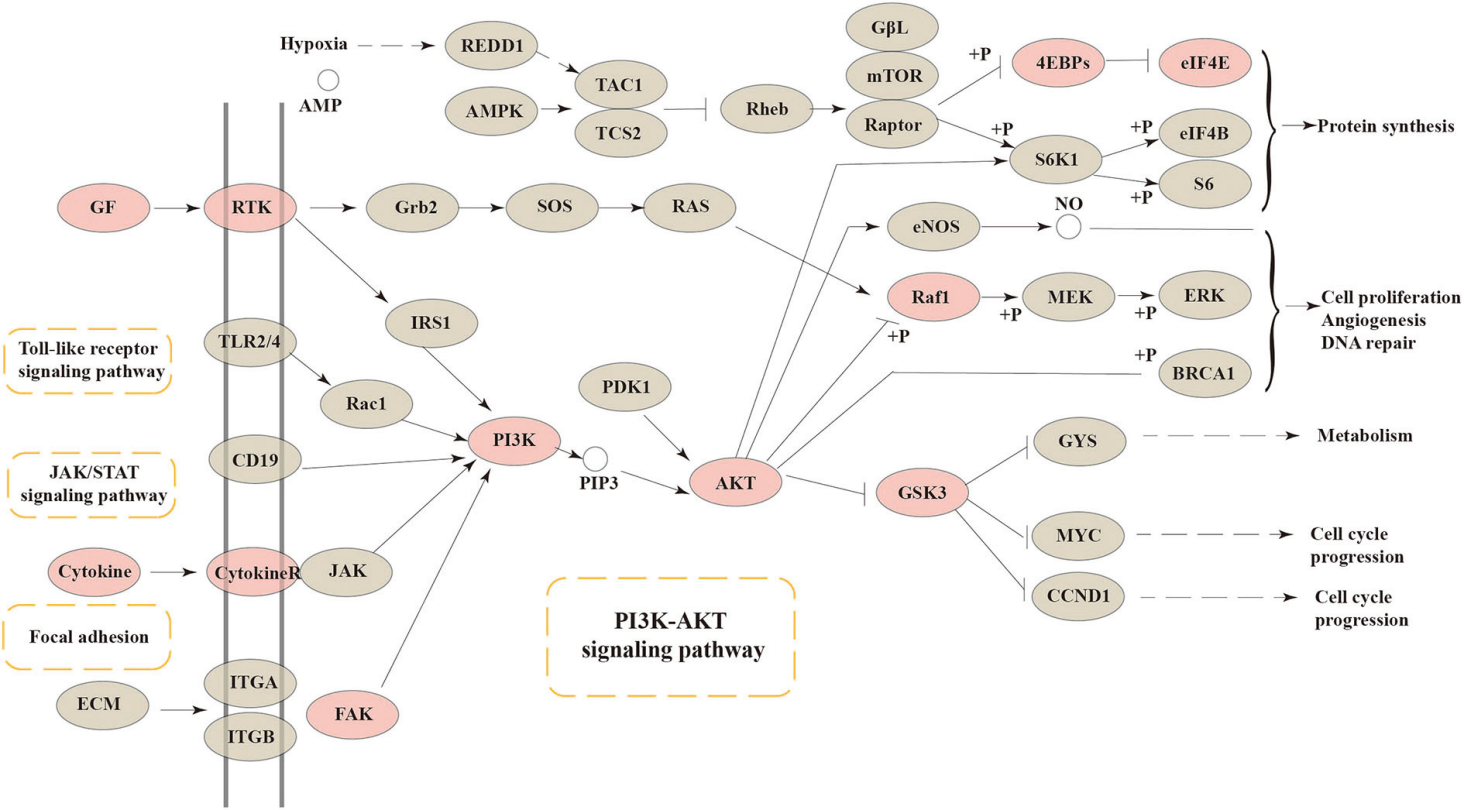
The Kyoto Encyclopedia of Genes and Genomes (KEGG) pathway enrichment analysis showed that various targets of the PI3K-AKT signaling pathway were tightly associated with the peptides' pharmaceutical actions. The red nodes represent the genes could be regulated or affected by fermented egg-milk peptides (FEMPs).

As a central and vital signal transduction pathway in many biological and physiological processes, the PI3K-Akt signaling pathway has a relationship with cell proliferation, morphology, apoptosis, migration, and synthesis (29). Besides, the PI3K-Akt signaling pathway has been confirmed to produce a relationship between intestinal health and colonic mucosa by previous references (30, 31). In our research, the connection between intestinal barrier function and the PI3K-Akt signaling pathway was confirmed again. The core targets of FEMPs could regulate and participate more in the PI3K-Akt signaling pathway. As Figure 6 depicts, cytokines, GF, RTK, FAK, PI3K, AKT, RAF1, GSK3, 4EBPs, and EIF4E were included in the PI3K-Akt signaling pathway. The result illustrated that the targets that FEMPs could regulate were distributed both upstream and downstream in the pathway, which could influence focal adhesion, protein synthesis, and cell cycle progression.

### Molecular docking

**Figure 7:**
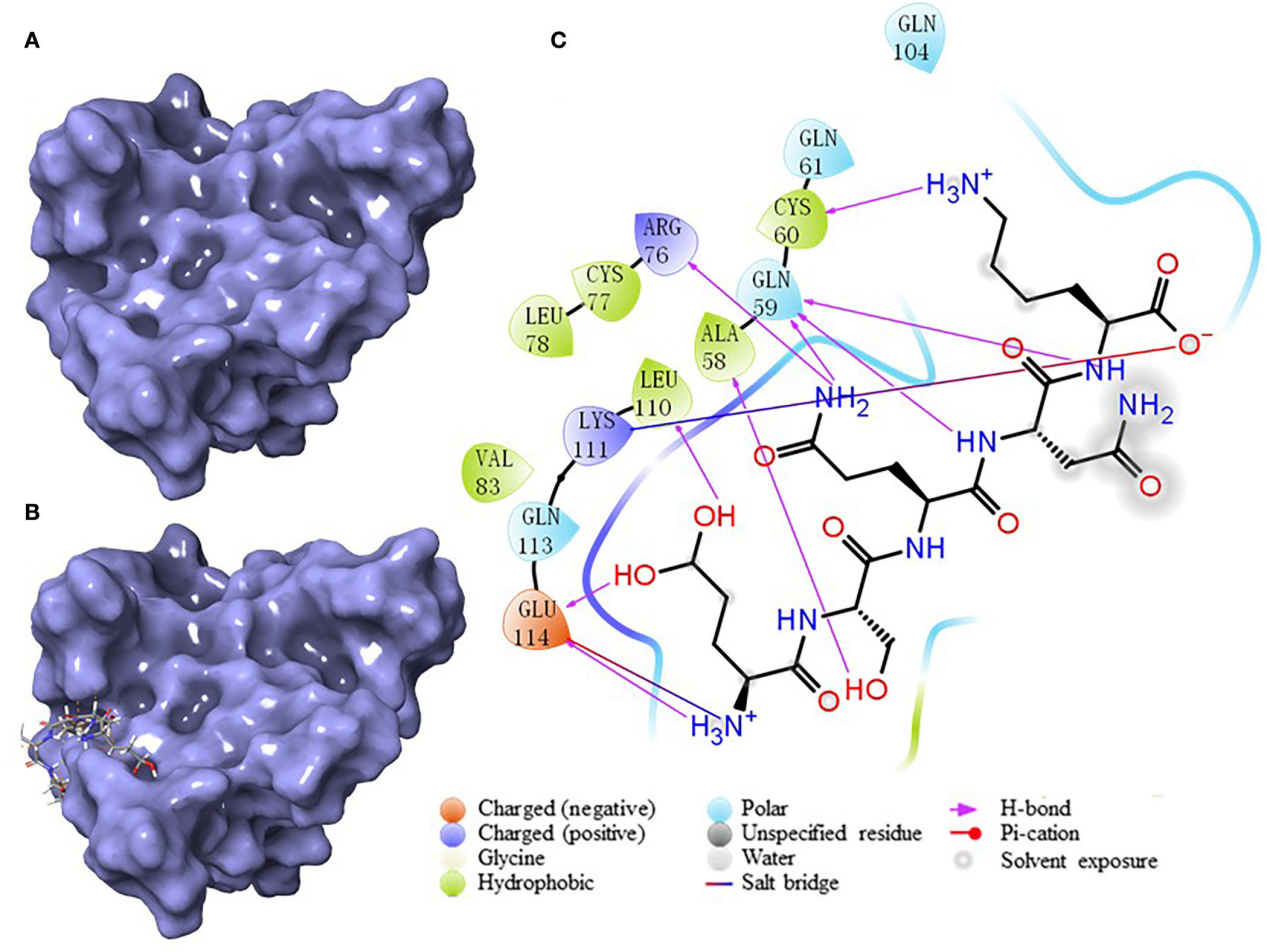
The results of molecular docking. **(A)** The crystal structure of AKT (PDB: 1UNQ). **(B)** The binding site location of the composite structure. **(C)** The interaction between ligand (ESQNK) and the receptor (AKT).

According to the result of the PPI network analysis, the target AKT revealed the highest degree, which indicated that this target might play a significant role in the PPI network and could affect other targets easily. Thus, the AKT target was chosen as the receptor in the molecular docking process. A widely known and powerful method, molecular docking, was developed here to demonstrate the interaction between the target and ligands. There have been many studies that have utilized molecular docking to define the interactions between different targets and peptides (19, 32, 33). The crystal structure of AKT (1UNQ, PDB doi: 10.2210/pdb1UNQ/pdb) was downloaded from the RCSB Protein Data Bank (http://www.rcsb.org/pdb) of the “Homo” organism (Figure 7A). The water molecules were removed during the molecular docking process. After the consummation of the calculation, the binding site was constructed. The ligands were set to bind at the site (Figure 7B). As the figure shows, the peptide was embedded in the active cavity at the docking site of the protein receptor and was fixed at the site by chemical force, forming a stable composite structure. The docking energy can numerically reflect how tightly the ligand binds to the receptor. Herein, the docking energy between peptides and the target was recorded. The result showed that FEMPs could bind to the receptor with a stable status and the top 15 FEMPs with their interaction information, as listed in Table 2. The docking energy ranged from −8.576 to −6.048 kcal mol^−1^. The peptide with the sequence ESQNK showed a docking energy of −8.576 kcal mol^−1^, which formed nine hydrogen bonds and two salt bridges in the combined interaction structure. For the interaction analysis of the combined structure, many residues contributed to the chemical bonds. The interactions between the peptide ESQNK and the target were visualized in Figure 7C. The interaction result revealed that GLU 114 and NH${}_{3}^{+}$ ion, LYS 111 and O^−^ ion formed one salt bridge, respectively. Six residues (GLU 114, LEU 111, ALA 58, GLN 59, CYS 60, and ARG 76) could produce hydrogen bonds in conformation with other atoms.

**Table 2:** The molecular docking energy between fermented egg-milk peptides (FEMPs) and AKT (The top 15).

**Peptides**	**Docking energy (kcal mol^−1^)**	**Hydrogen bond**	**Salt bridge**
ESQNK	−8.576	9	2
ESIIN	−7.749	7	0
DEY	−7.140	7	1
DSNVN	−7.066	8	1
DEK	−7.044	7	2
ENR	−6.757	8	1
YAEER	−6.638	6	1
EGTT	−6.596	8	0
QWQV	−6.576	7	1
ESN	−6.419	6	1
LYAEER	−6.354	6	0
VEDI	−6.267	5	2
DGGQA	−6.210	8	1
AEERY	−6.146	6	1
DSTR	−6.048	8	1

## Discussion

In recent years, the intestine's role as a protective organ of the body has brought it to the forefront of medical research. In addition, intestinal immune capacity has become a hot and well-known topic in recent research (34, 35). As a crucial part of the intestinal tract, the intestinal mucosal barrier contributes greatly to intestinal digestion, absorption, and barrier protection. More and more evidence has confirmed that the intestinal barrier not only acts as a medium for absorbing and exchanging nutrients and other substances but also plays an essential role in keeping external antigens and harmful armamentariums away from entering the body (1). Intestinal was also related closely to the immune system. Previous studies have reported that the intestinal mucosal barrier was associated with the toll-like receptor (TLR) signal pathway, which represented the significant processes of the immune system and its functions (36). Tight junctions, made up of Zonula occludens 1 (ZO-1), claudin, and occluding, were reported to contribute beneficially to the structure and function of the intestinal epithelial cells in the colon (37). The previous paper demonstrated that the disorder of ZO-1 might cause the intestinal barrier to weaken and become dysfunctional, aggravating the inflammation of the intestine (38). MUC-2, one of a family of different gel-forming mucins, was considered an important component of the loose outer layer and the inner mucus layer that is firmly adhered to epithelial cells (39). It could provide nutrients to the intestinal epithelial cells and intestinal flora in the colon, contributing to intestinal protection. Based on the significance of the two vital proteins, they were always seen as the key factors related to the intestinal barrier and intestinal function. In the current study, these two proteins were used as the evaluation indicators to assess the damage to the intestinal barrier, and DSS-induced colitis was performed here.

Bioactive peptides derived from the raw material or other food ingredients were reported as having intestinal protective functions and features. Our previous research found that fermented egg-milk peptides could alleviate intestinal inflammation symptoms and establish the gut microbiota in colitis mice (15). However, the protective function of the damaged intestinal tissue barrier and the underlying biological mechanism are not clear now. In the current study, the core idea of network pharmacology was utilized to develop the analysis of FEMPs on the potential targets related to the intestinal barrier. Network pharmacology, a widely used method with the idea of “multi-targets and multi-pathways,” would naturally become a widely used strategy and unbiased method for developing the underlying and potential mode of action of natural foods and their functional ingredients (40). We mapped the interaction figure of possible interactions between the targets not only to investigate the application potential of functional ingredients but also to develop their possible interactions, thereby increasing the speed of the development process (41). FEMPs, with their characteristic multicomposition, were suitable for the application of network pharmacology. Herein, the assessment model of colitis mice and bioinformatics methods such as network pharmacology and molecular docking technology were co-utilized to find out the pathological mechanism FEMPs could exert on the targets. It could provide a new view for intestinal barrier damage restoration, and colonic disease treatment broadens the horizon of egg products' functional applications.

The expression and location of key proteins such as ZO-1 and MUC-2 could influence the function and condition of the colon. To further demonstrate the effect of FEMPs on the reconstruction of the two key proteins, the commonly used method of evaluating intestinal damage, immunofluorescence analysis, was performed here. At present, many studies have used immunofluorescence to analyze the location, distribution, and function of such proteins in the intestinal. Bian and his partner used immunofluorescence analysis to clarify the effect of *Akkermansia muciniphila* on two important evaluation markers of the intestinal barrier, ZO-1 and Occludin (42). Kim performed immunofluorescence to analyze the influence of short-chain fatty acids (SCFAs) on ZO-1 in the colon tissue of inflammatory mice (43). In addition to using immunofluorescence analysis, in this paper, we also employ western blotting to detect the expression of these two vital proteins. Herein, the results showed that FEMPs could enhance the expression of ZO-1 and MUC-2, which benefited the construction of an intestinal barrier.

Manu studies have shown that bioactive peptides can improve the intestinal barrier and that bioactive peptides can regulate the expression of ZO-1 and MUC-2. Zou has researched the protective function of the intestinal tight junction with tissue factor-related peptides (44). The effect of shrimp peptide on the intestinal barrier of cyclophosphamide-treated mice was detected, and the results revealed the peptide could increase tight-junction-associated proteins (45). Our study also demonstrated the beneficial influence of FEMPs on damaged intestinal barrier repair and construction in colitis mice induced by DSS, making the study of bioactive peptides in the intestinal barrier more complete and holistic.

Based on the idea of multi-targets and multi-pathways, network pharmacology, a novel method used to analyze the pharmacological effects of multi-ingredient compounds, was performed here to further define the regulation of FEMPs. PPI networks, GO, and KEGG analyses were developed in this research to investigate the underlying biological mechanisms. The PPI network enrichment results revealed that AKT, CASP3, SRC, HPGDS, and MMP9 play vital roles, and many references confirmed the significance of such targets. Akt acted as a central and core part in the regulation of cellular anti-apoptosis effect on a majority of body diseases, which might influence the proliferation and metabolism of intestinal epithelial cells (46). CASP3 is a key enzyme that regulates the process of apoptosis. SRC might regulate cell growth, differentiation, and survival through unequal signal transduction, which affects cell adhesion, migration, and invasion. Such significant targets were enriched in the PPI network, which indicated that the interaction of the target set of FEMPs was complete and consummate. GO analysis (BP) revealed that the targets could regulate the endopeptidase activity associated with the apoptotic process, glutathione metabolic process, and cysteine-type endopeptidase activity regulation. A previous paper demonstrated the relationship between the biological process and intestinal epithelial cells (47). In the part of CC, the biological function included the tertiary granule lumen and ruffle membrane. The MF results indicated that kinds of kinase activity were mentioned. These results demonstrate that FEMPs can be a functional ingredient applied to intestinal damage by targeting the kinase, the same as Yan's paper (48). KEGG analysis revealed the core targets mostly participated in the PI3K-Akt pathway, focal adhesion, the TLR signaling pathway, the MAPK signaling pathway, the EGFR tyrosine kinase inhibitor resistance, and the VEGF signaling pathway. The aforementioned pathways were related to the intestinal barrier and colonic health, which could regulate the vital biological processes of the intestinal epithelial cells.

Previous references have reported that the PI3K/Akt pathway could have a relationship with the proliferation of transformed intestinal epithelial cells (49, 50). Bian and his partners also demonstrated that the PI3K/Akt signaling pathway could affect the distribution of ZO-1 in epithelial cells (51). Our results were consistent with the above papers. TLR, as former research described, could mediate the process of intestinal barrier breakdown (52). Our KEGG results also demonstrated the relationship between TLR and the intestinal barrier. MAPK signaling pathway, according to Zheng's paper, was linked to intestinal barrier disruption and colonic inflammation occurred in the colon (53). In brief, we speculated that such a pathway could occur in relation to the key proteins ZO-1 and MUC-2 in the colon and then play important roles in the regulation of intestinal barrier function.

To further research the interaction between FEMPs and the key protein, molecular docking, a powerful and widely used method, was developed in the current study. It could be a significant strategy based on the computer calculation of receptor-ligand interaction for predicting peptides with the core target (19). Akt, with the highest degree in our study, was used as the receptor in the molecular docking part. Akt could affect the transcriptional regulation of cellular genetic information and cell survival, metabolism, differentiation, growth, migration, and angiogenesis in the bodies. Besides, it has a close relationship with the intestinal barrier, and its activity regulation was seen as an effective way to enhance it (54). A reliable paper demonstrated the regulation style of “Akt-in” peptides, and their binding sites were described in this research (46). As we expected, the FEMPs could bind to the receptor with a lower docking score and less energy in our study, indicating that FEMPs could combine with the receptor in a stable condition.

Moreover, hydrogen bonds and salt bridges play vital roles in maintaining the structure of the ligand receptor. According to the results, we inferred that FEMPs might be able to closely chimera with Akt, thus occupying its active site and affecting the enzyme activity. This may enhance the distribution and expression of tight junction proteins in intestinal epithelial cells and result in the repair of the damaged intestinal barrier.

## Conclusion

In this study, the protective function and underlying targets of FEMPs on the damaged intestinal barrier were investigated based on immunofluorescence analysis, western blot, and network pharmacology. The potential mechanism appeared to affect the peptidase activity in the apoptotic process, serine/tyrosine kinase activity, and cellular metabolism-related pathways such as the PI3K-Akt signaling pathway, TLR signaling pathway, and MAPK signaling pathway. Besides, FEMPs could combine the key protein Akt with hydrogen bonds and salt bridges. Based on the analysis of multiple targets and multiple pathways, this study could offer evidence for the regulation of peptides on the damaged intestinal barrier and provide a new perspective and ideas for the research of bioactive peptides and their biological functions.

## Data availability statement

The original contributions presented in this study are included in the article/Supplementary material, further inquiries can be directed to the corresponding author.

## Ethics statement

The animal study was reviewed and approved by Animal Ethics Committee of Jilin University (Approval No.20200483).

## Author contributions

SL: writing—original draft preparation. QY, XD, SL, and XL: data curation and formal analysis. ZD, TZ, and XS: writing—reviewing and editing. MX: sample collection. JL and FP: supervision. TZ, JL, and FP: conceptualization. All authors contributed to the article and approved the submitted version.
