# Supplementary material for: Protective effects and potential mechanisms of fermented egg-milk peptides on the damaged intestinal barrier
Source: Front Nutr. 2022 Dec 7;9:1068877. doi: 10.3389/fnut.2022.1068877 (PMC9767966; doi:10.3389/fnut.2022.1068877)
Supplement: Supplementary file 1 [file Table_1.docx]

Table.S1 The targets with the node degree larger than 10 in protein-protein interaction network (PPI)

| Targets | Node degree | Targets | Node degree |
| --- | --- | --- | --- |
| AKT1 | 31 | HGF | 14 |
| CASP3 | 26 | RAF1 | 14 |
| SRC | 23 | PTPN1 | 13 |
| HPGDS | 20 | AKR1B1 | 12 |
| MMP9 | 19 | PIK3R1 | 12 |
| GSR | 15 | XIAP | 12 |
| PTK2 | 15 | IL2 | 11 |
| GSK3B | 14 | G6PD | 10 |
| GSTP1 | 14 | GSTM1 | 10 |

Table.S2 GO analysis

| **GO term** | **subgroup** | **Enrichment score** |
| --- | --- | --- |
| glutathione metabolic process | Biological process | 50.74 |
| cellular modified amino acid metabolic process | Biological process | 35.69 |
| sulfur compound metabolic process | Biological process | 22.47 |
| transmembrane receptor protein tyrosine kinase signaling pathway | Biological process | 18.64 |
| peptide metabolic process | Biological process | 14.27 |
| enzyme-linked receptor protein signaling pathway | Biological process | 12.46 |
| negative regulation of cysteine-type endopeptidase activity involved in apoptotic process | Biological process | 52.79 |
| negative regulation of cysteine-type endopeptidase activity | Biological process | 47.82 |
| negative regulation of apoptotic signaling pathway | Biological process | 22.72 |
| negative regulation of extrinsic apoptotic signaling pathway | Biological process | 41.91 |
| cellular amide metabolic process | Biological process | 9.53 |
| icosanoid metabolic process | Biological process | 35.35 |
| regulation of cysteine-type endopeptidase activity involved in apoptotic process | Biological process | 22.77 |
| response to peptide | Biological process | 13.17 |
| tertiary granule lumen | Cellular component | 31.68 |
| ficolin-1-rich granule lumen | Cellular component | 23.42 |
| ruffle membrane | Cellular component | 17.96 |
| extrinsic component of cytoplasmic side of plasma membrane | Cellular component | 17.60 |
| ficolin-1-rich granule | Cellular component | 15.70 |
| cytoplasmic side of membrane | Cellular component | 14.23 |
| cytoplasmic side of plasma membrane | Cellular component | 13.43 |
| leading edge membrane | Cellular component | 13.20 |
| tertiary granule | Cellular component | 10.62 |
| extrinsic component of plasma membrane | Cellular component | 10.25 |
| antioxidant activity | Molecular function | 20.50 |
| protein serine/threonine/tyrosine kinase activity | Molecular function | 10.42 |
| phosphotransferase activity, alcohol group as acceptor | Molecular function | 7.75 |
| kinase activity | Molecular function | 7.16 |
| protein kinase activity | Molecular function | 8.22 |
| hormone binding | Molecular function | 20.03 |
| protein serine kinase activity | Molecular function | 6.43 |
| protein serine/threonine kinase activity | Molecular function | 5.40 |
| cysteine-type endopeptidase regulator activity involved in apoptotic process | Molecular function | 43.55 |
| peptidase regulator activity | Molecular function | 7.57 |
| metallopeptidase activity | Molecular function | 21.97 |

Table.S3 KEGG analysis

| **Class** | **Description** | **Enrichment** | **p-value** | **count** |
| --- | --- | --- | --- | --- |
| KEGG | PI3K-Akt signaling pathway | 16.40482 | 4.41E-10 | 10 |
| KEGG | Lipid and atherosclerosis | 24.30966 | 1.12E-10 | 9 |
| KEGG | Focal adhesion | 23.11366 | 1.96E-09 | 8 |
| KEGG | Proteoglycans in cancer | 22.66266 | 2.29E-09 | 8 |
| KEGG | EGFR tyrosine kinase inhibitor resistance | 51.45716 | 7.77E-11 | 7 |
| KEGG | C-type lectin receptor signaling pathway | 39.08765 | 5.51E-10 | 7 |
| KEGG | Insulin signaling pathway | 29.67238 | 3.82E-09 | 7 |
| KEGG | Yersinia infection | 29.67238 | 3.82E-09 | 7 |
| KEGG | Kaposi sarcoma-associated herpesvirus infection | 20.9542 | 4.24E-08 | 7 |
| KEGG | Diabetic cardiomyopathy | 20.0252 | 5.79E-08 | 7 |
| KEGG | Rap1 signaling pathway | 19.35769 | 7.3E-08 | 7 |
| KEGG | Human cytomegalovirus infection | 18.06718 | 1.17E-07 | 7 |
| KEGG | MAPK signaling pathway | 13.82692 | 7.1E-07 | 7 |
| KEGG | VEGF signaling pathway | 59.05737 | 8.13E-10 | 6 |
| KEGG | ErbB signaling pathway | 40.99276 | 7.63E-09 | 6 |
| KEGG | Prostate cancer | 35.92149 | 1.7E-08 | 6 |
| KEGG | Endocrine resistance | 35.55495 | 1.81E-08 | 6 |
| KEGG | Relaxin signaling pathway | 27.01073 | 9.37E-08 | 6 |
| KEGG | Apoptosis | 25.62048 | 1.28E-07 | 6 |
| KEGG | mTOR signaling pathway | 22.3358 | 2.89E-07 | 6 |
| KEGG | Hepatitis B | 21.50855 | 3.61E-07 | 6 |
| KEGG | Prolactin signaling pathway | 41.48077 | 1.38E-07 | 5 |
| KEGG | Colorectal cancer | 33.76342 | 3.89E-07 | 5 |
