# Supplementary figures and images for: Protective effects and potential mechanisms of fermented egg-milk peptides on the damaged intestinal barrier
Source: Front Nutr. 2022 Dec 7;9:1068877. doi: 10.3389/fnut.2022.1068877 (PMC9767966; doi:10.3389/fnut.2022.1068877)

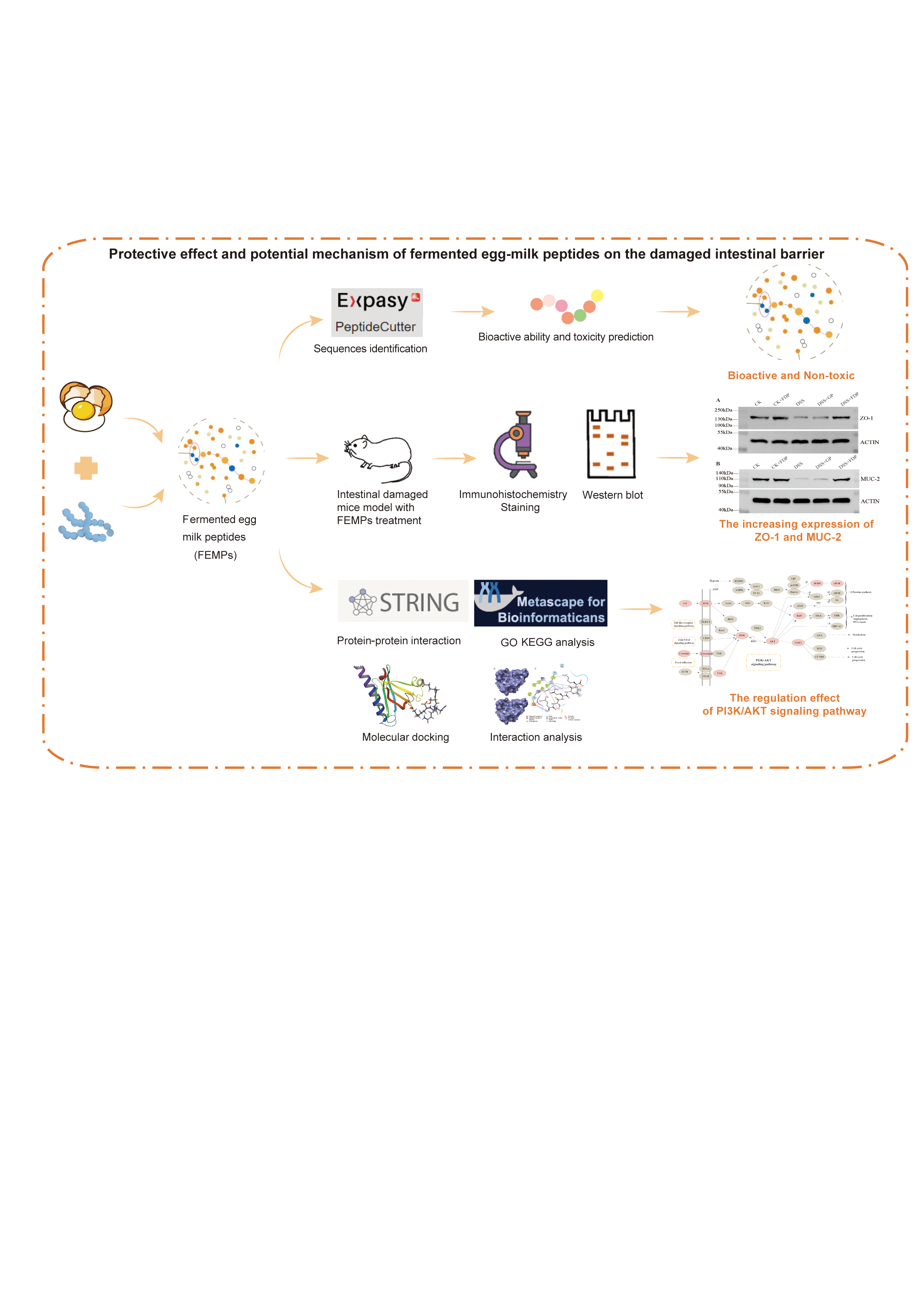

Supplement: Supplementary file 2 [file Image_1.TIF]
